# Defect-Mediated Crystallization of the Particulate TiO_2_ Photocatalyst Grown by Atomic Layer Deposition

**DOI:** 10.1021/acs.jpcc.4c07091

**Published:** 2024-12-19

**Authors:** Bela D. Bhuskute, Harri Ali-Löytty, Jesse Saari, Arto Hiltunen, Tero-Petri Ruoko, Turkka Salminen, Mika Valden

**Affiliations:** † Surface Science Laboratory, Faculty of Engineering and Natural Sciences, Tampere University, P.O. Box 692, FI-33014 Tampere, Finland; ‡ Liquid Sun Ltd., Tekniikankatu 1, FI-33720 Tampere, Finland; § Archipelago Research Institute, Biodiversity Unit of the University of Turku, 20014 Turku, Finland; ∥ Spectroscopy and Light-Active Materials, Faculty of Engineering and Natural Sciences, Tampere University, P.O. Box 692, FI-33014 Tampere, Finland; ⊥ Tampere Microscopy Center, Tampere University, P.O. Box 692, FI-33014 Tampere, Finland

## Abstract

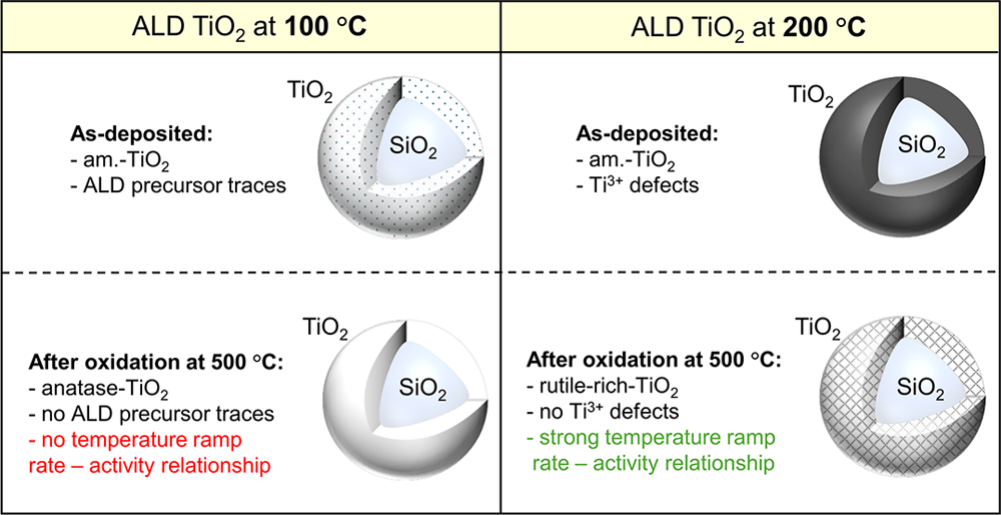

Nanopowders or films of pure and mixed oxides in nanoparticulate form have gained specific interest due to their applicability in functionalizing high-surface-area substrates. Among various other applications, our presented work primarily focuses on the behavior of TiO_2_ as a photocatalyst deposited by atomic layer deposition (ALD) on a quartz particle. The photocatalytic activity of TiO_2_ on quartz particles grown by ALD was studied in terms of ALD growth temperature and post-treatment heating rate. Amorphous TiO_2_ thin films (30 nm) were grown from tetrakis(dimethylamido)titanium (TDMAT) at 100 and 200 °C on quartz particles (0.35–3.5 μm) and crystallized using oxidative heat treatment at 500 °C with variable heating rates. The growth temperature was found to affect the TiO_2_ defect structure: TiO_2_ grown at 200 °C is black due to Ti^3+^ defects, whereas the film grown at 100 °C is white but contains some traces of the TDMAT ALD precursor. During the oxidative heat treatment, precursor traces desorbed and Ti^3+^ defects were oxidized. ALD TiO_2_ grown at 100 °C crystallized as anatase, whereas the rutile-to-anatase ratio of 200 °C grown TiO_2_ increased with the heating rate. The hydrogen production rate of mixed-phase TiO_2_ was found to outperform that of anatase TiO_2_.

## Introduction

Sustainable management of energy resources and protection of the environment are among the most important issues that our society must address. Solar water splitting (SWS) for hydrogen production^1^ offers a scientifically interesting, clean, and environmentally friendly alternative to fossil fuels for future energy needs. The discovery of the Honda–Fujishima effect has indeed expanded research and development opportunities in solar water splitting and photocatalysis.^2^

Titanium dioxide (TiO_2_) as a photocatalyst has tremendous potential to address these challenges by harnessing solar energy for various applications such as water purification, air pollution control, and solar energy conversion, leading us to a more sustainable and greener future.^3,4^ With its exceptional properties, including high chemical stability, low toxicity, and strong oxidative capabilities, titanium dioxide has attracted considerable attention in the field of photocatalysis.^3,5−200^ The crystal structure and phase of TiO_2_ play an important role in determining its photocatalytic activity, and hence, it can be used in many photocatalytic processes. Therefore, understanding and controlling the crystallization of TiO_2_ is very important to optimize its performance for different applications such as photocatalysis, sensors, solar cells, energy storage devices, etc.

In the field of photocatalysis, nanosized TiO_2_ still remains the most promising material due to its exceptional properties.^8−10^ The prevailing preference for particulate photocatalysis is the commercially obtainable Degussa P25 powder (average particle size of 25 nm),^11−13^ which is composed of a combination of anatase, rutile, and amorphous forms of TiO_2_. P25 has optimum dimensions in terms of carrier diffusion length and surface-to-bulk ratio. However, the anatase-to-rutile-amorphous ratio of P25 depends on processing and can have a significant effect on performance.

In this study, we have deposited a titanium dioxide shell on a particulate silica support by the atomic layer deposition (ALD) technique to create SiO_2_–TiO_2_ core–shell particles in order to demonstrate controlling of the TiO_2_ phase structure by ALD growth temperature and post-treatment heating treatment. It is globally recognized that ALD is an optimal technique to conformally deposit photocatalyst materials on large-surface-area substrates with the intended composition and thickness.^14−16^ Extensive research has also shown that postdeposition annealing plays a vital role in influencing the crystallization of TiO_2_ and consequently improving its photocatalytic performance.^17^ The heating rate during the annealing process can affect the diffusion of impurities, nucleation, and growth of TiO_2_ crystals, leading to variations in their size, shape, and crystalline structure.

In order to enhance the photocatalytic efficiency, we investigated how the crystal structure and phase of titanium dioxide are modified by varying the ALD growth temperature and rapid thermal annealing (RTA) in order to transform the TiO_2_ crystal structure from anatase to a mixture of anatase–rutile phase on SiO_2_ support. We further investigated the impact of heating rates on reaction kinetics, including activation energies for desorption and crystallization, which subsequently affected the rutile-to-anatase ratio. Following that, photocatalytic tests were carried out to demonstrate the influence of the TiO_2_ phase transformation on photocatalytic efficiency.

## Experimental Section

### Substrates

Quartz particles (SiO_2_, 0.35–3.50 μm particle size, BCR066, BCR certified reference material, Sigma-Aldrich) were used as a substrate in all of the experiments. Relatively large particle size, c.f., size of benchmark photocatalyst P25, facilitated the growth of conformal ALD coating but resulted in low apparent activity if normalized to the sample mass.

### Synthesis of SiO_2_–TiO_2_ Core–Shell Particles by Atomic Layer Deposition

A uniform and thin layer of quartz particles was meticulously applied onto the surface of a Petri dish, ensuring an even distribution across the entire area. TiO_2_ deposition on these quartz particles was conducted employing a Picosun Sunale ALD R-200 Advanced reactor at growth temperatures of 100 and 200 °C. The chosen precursors for this process were tetrakis(dimethylamido)titanium(IV) (TDMAT, Ti(N(CH_3_)_2_)_4_) and Milli-Q type 1 ultrapure water. The selection of precursors for 30 nm thick ALD TiO_2_, the specific ALD procedure utilized, and the determination of growth temperatures of 100 and 200 °C were based on a comprehensive examination and analysis of the research conducted by Saari et al.^18^ To achieve 30 nm TiO_2_ layer thickness, 480 and 870 ALD cycles were used at growth temperatures of 100 and 200 °C, respectively. A single ALD cycle comprised a 1.6 s pulse of TDMAT and a subsequent 0.1 s H_2_O pulse. In between each pulse, a 0.6 s purging period was utilized to eliminate the excess precursor.

### Rapid Thermal Annealing (RTA)

To perform rapid thermal annealing in ambient air, the TiO_2_-deposited quartz particles were inserted into a tube furnace that had been preheated to 500 °C with the temperature increasing rapidly at a ramping rate exceeding 1000 °C/min as monitored by a thermocouple. The annealing process continued for a duration of 45 min. Following the heat treatment, the samples were removed from the tube furnace and subjected to passive cooling, allowing them to naturally reach ambient temperature.

### UV/vis Spectrophotometry Analysis

The optical properties of the particles were assessed by utilizing a PerkinElmer LAMBDA 1050 UV/vis/NIR spectrophotometer, incorporating an integrating sphere detector. In order to assess the absorbance of the samples, diffuse reflectance spectra were obtained across the wavelength range of 300–600 nm for powder films prepared by drop-casting aqueous particle solution onto glass substrates.

Absorbance (*A*) was calculated from reflectance (*R*) by the Kubelka–Munk approach: *A* = *f*(*R*) = (1 – *R*)^2^/2*R*. Band gap values were determined by the Tauc plot analysis assuming the indirect allowed band gap for TiO_2_.

### Differential Scanning Calorimetry (DSC) and Thermogravimetric (TG) Analysis

The oxidation process of these samples was carried out in an oxygen atmosphere under 60 mL/min flow using a Differential Scanning Calorimetry instrument (Mettler Toledo DSC 1), employing different heat ramping rates ranging from 5 to 100 °C/min to capture any phase transitions or thermal events.

Thermogravimetric analysis (TGA) was performed with a Mettler Toledo TGA850 at a ramp rate of 40 °C/min.

### Raman Analysis

Using a Renishaw inVia Qontor Raman microscope, the Raman spectra of the samples were measured with a 532 nm laser employed for the measurements.

### Photocatalytic Hydrogen Production Test

In order to evaluate the photocatalytic performance, 20 mg of catalyst particles were taken in a 50 mL quartz photoreactor (quartz round-bottom flask, QRB, total dead space 75 mL) to study the photocatalytic hydrogen production reaction. Particle slurry was prepared by mixing the catalyst particles with 40 mL of 25% v/v aq methanol solution. The reactor was illuminated by a 300 W Xe lamp (MAX-350 equipped with a UV–vis mirror module producing a spectrum in the range of 300–600 nm, Asahi Spectra Co., Ltd.). Gas chromatography was subsequently utilized to conduct the H_2_ production analysis. We followed the detailed experimental procedure outlined in our previous article to evaluate the photocatalytic performance of these photocatalyst particles.^7^

## Results and Discussion

**Figure 1:**
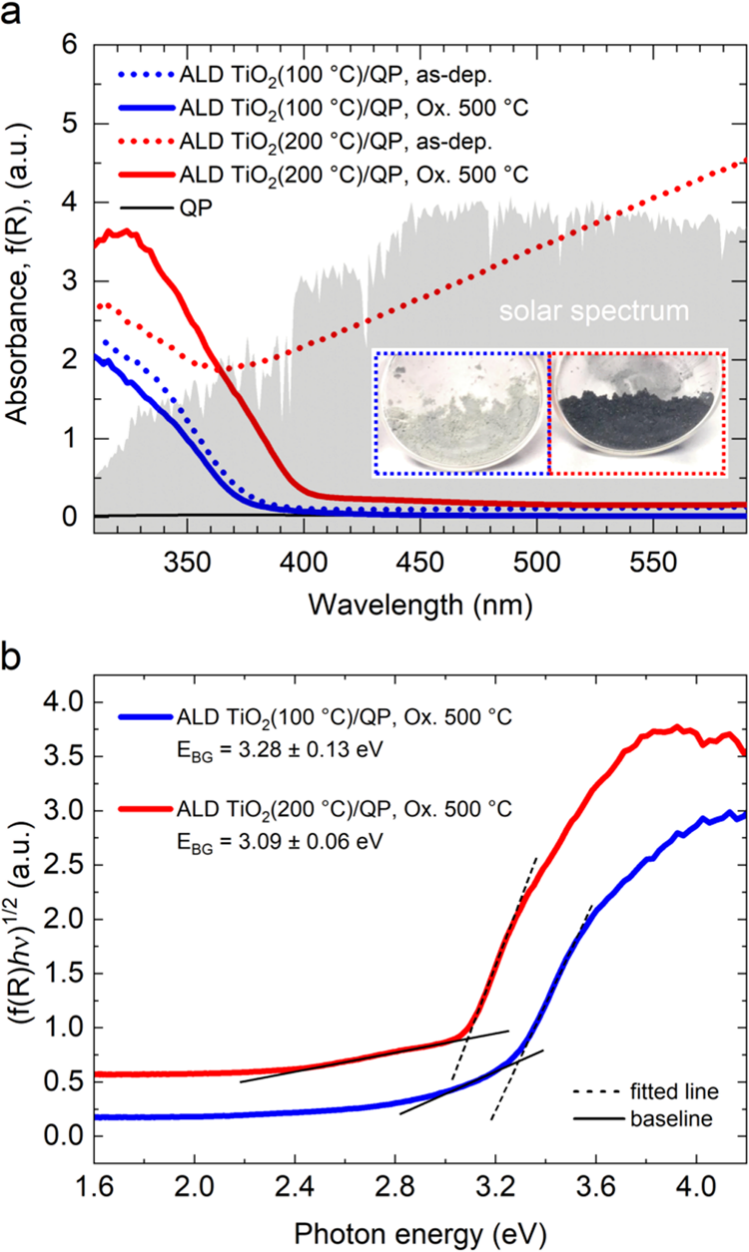
(a) Absorbance of samples as determined from the diffuse reflectance measurement. (b) Tauc plot analysis. Band gap values are determined as the intersection of the linear fits to the absorption edge and the baseline. The band gap error value is derived from the standard deviation of the linear fits using the propagation of error principle.

To obtain a comprehensive understanding of how ALD growth temperature and post-oxidative heat treatment at 500 °C impact TiO_2_ crystallization under ambient air, we analyzed the absorbance of these SiO_2_–TiO_2_ core–shell particles both before and after undergoing oxidative heat treatment at a temperature of 500 °C. Figure 1a depicts the absorption spectra obtained from the diffuse reflectance spectra recorded using a UV/vis spectrophotometer. At a low deposition temperature of 100 °C, the as-deposited amorphous (am.-TiO_2_) particles display a white physical appearance. However, a transition occurs as the deposition temperature increases to the higher value of 200 °C, resulting in the particles acquiring a black color giving rise to the strong absorbance through the visible wavelength range. This absorption can be assigned to Ti^3+^ defects within the as-deposited TiO_2_ films grown by ALD.^19^ However, particles subjected to annealing in air at 500 °C show a lack of visible light absorption. In all of the presented samples, a strong band gap absorption edge was observed for the <390 nm range. The displacement of the absorption edge position indicates the existence of distinct band gap values. The results obtained from the Tauc plot analysis presented in Figure 1b highlight a noteworthy trend in the oxidative heat-treated samples, showing a decrease in the band gap values from 3.28 ± 0.13 to 3.09 ± 0.06 eV with the increase in ALD growth temperatures from 100 to 200 °C, respectively, corresponding to anatase and rutile phases, respectively. Plain quartz particles (QP, SiO_2_, band gap of 9 eV) had no absorbance in the visible wavelength range.

**Figure 2:**
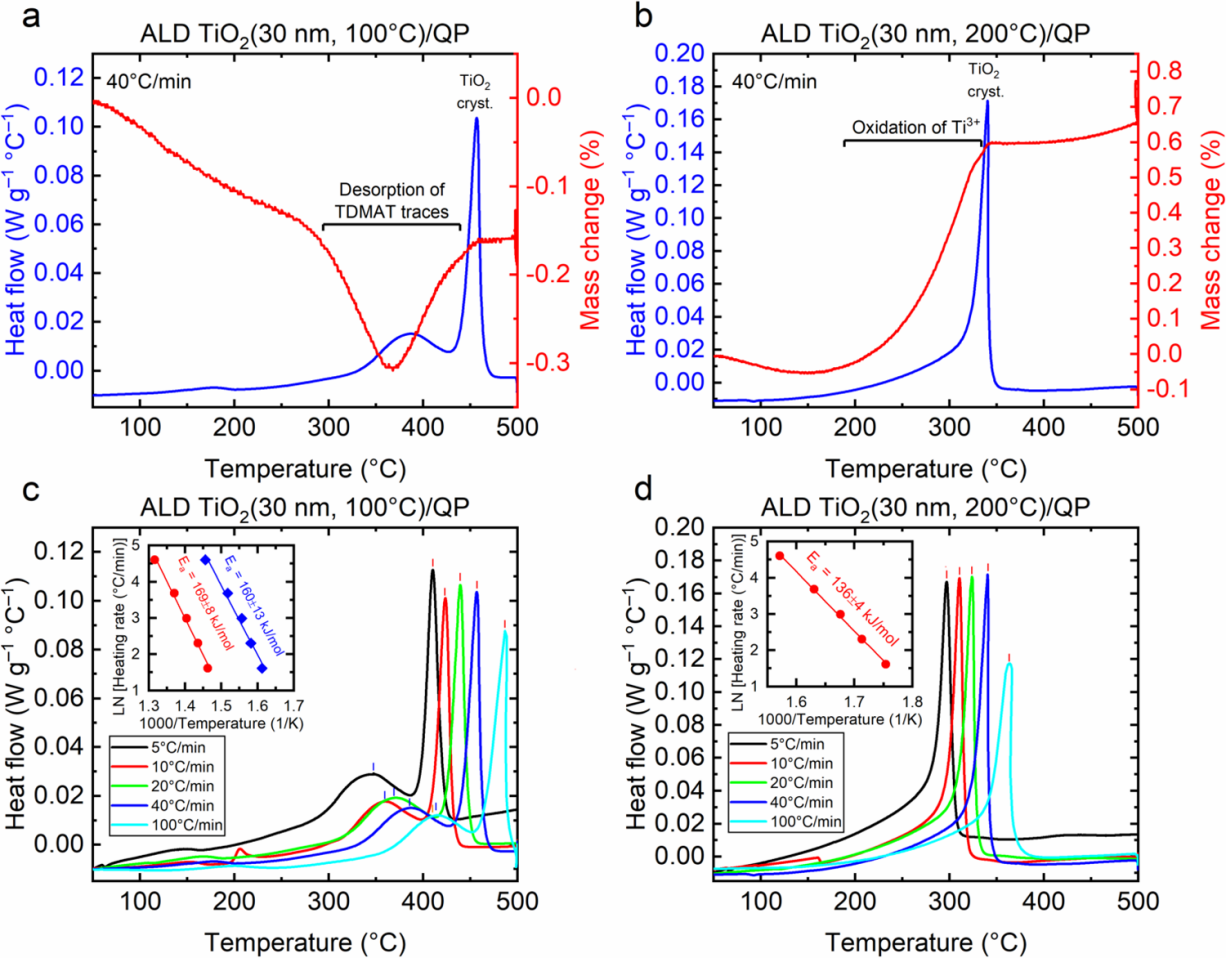
Differential scanning calorimetry (DSC) and thermogravimetric (TG) analysis: DSC and TG thermograms measured at 40 °C/min for ALD TiO_2_ particles grown at (a) 100 °C and (b) 200 °C. DSC thermograms measured at 5–100 °C/min for ALD TiO_2_ particles grown at (c) 100 °C and (d) 200 °C. Insets in (c) and (d) show activation energy analysis for the observed kinetic processes: desorption and crystallization for the TiO_2_ grown at 100 °C and only crystallization for the TiO_2_ grown at 200 °C.

Differential scanning calorimetry (DSC) and thermogravimetric analysis (TGA) were conducted to investigate the effect of ALD growth temperature and post-heating treatment ramp rate on the crystallization mechanism of ALD TiO_2_. Heat flow and weight loss behavior of the SiO_2_–TiO_2_ particles were measured at varying ramping rates between 5 and 100 °C/min. Sharp exothermic peaks in the thermograms that do not involve mass change indicate crystallization. Figure 2a demonstrates the DSC and TGA curves for the TiO_2_ particles grown at 100 °C and post-heating treatment at a ramping rate of 40 °C/min in an oxygen atmosphere. The thermograms exhibited two distinct exothermic kinetic processes. The first process involves the desorption of TDMAT precursor traces seen as a distinct weight loss step in the TGA curve at approximately 370 °C, which takes place before the second process of crystallization, where the amorphous TiO_2_ transforms into a crystalline phase. In contrast, DSC thermograms of ALD TiO_2_ grown at 200 °C (Figure 2b) show only one sharp exothermic peak that can be attributed to crystallization. For both ALD growth temperatures, crystallization precedes an increase in mass due to the incorporation of oxygen into the lattice. The mass increase was stronger for the sample grown at 200 °C containing a higher concentration of Ti^3+^ defects that oxidized to Ti^4+^ in the process.

The analysis of the activation energies for the kinetic processes is depicted in Figure 2c,d. The activation energy of crystallization was 136 ± 4 kJ/mol for the samples grown at 200 °C and 169 ± 4 kJ/mol for the sample grown at 100 °C. The activation energy for the desorption of TDMAT traces was 160 ± 13 kJ/mol, within the error bars with the activation energy for crystallization. It can be concluded that ALD precursor traces increase the activation energy for TiO_2_ crystallization. A practical implication is the higher temperature required for crystallization.

**Figure 3:**
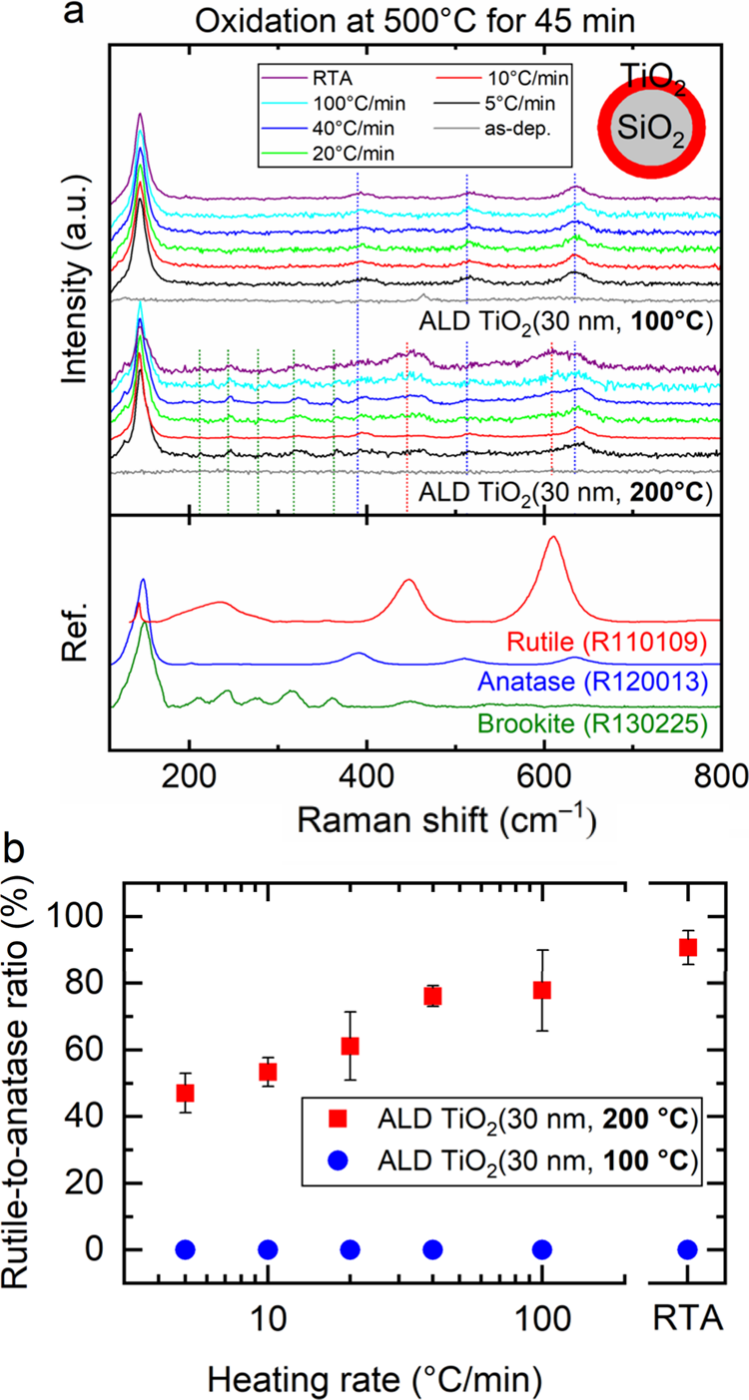
(a) Stacked Raman spectra illustrating the progression from a state of pure anatase to a combination of anatase–rutile phase mixtures with respect to ALD growth temperature. (b) Rutile-to-anatase ratio for particles grown at 100 and 200 °C with respect to heating rate for oxidative PDA. Error bars represent the standard deviation of peak areas.

The Raman spectra were measured to provide insights into the crystal structure of deposited 30 nm thick ALD TiO_2_ grown at 100 and 200 °C, crystallized at 500 °C by two processes: controlled heating ramp between 5 and 100 °C/min and rapid thermal annealing (RTA) at approximately 1000 °C/min. Figure 3a discloses that as-deposited ALD TiO_2_ grown at 100 and 200 °C shows an amorphous nature. However, after oxidative annealing at 500 °C, all of the ALD TiO_2_ samples grown at 100 °C exhibit only Raman peaks characteristic of the anatase phase. In contrast, ALD TiO_2_ samples grown at 200 °C show more complex spectra of multiple crystalline phases.

The peaks confirm the presence of the anatase phase, while additional peaks in the spectra indicate the coexistence of rutile and brookite phases. Rapid heating rates promote the crystallization of rutile, as evidenced by intensified rutile-specific Raman peaks for TiO_2_ grown at 200 °C. Prior studies on TiO_2_ thin films have indicated that the abrupt transformation from am.-TiO_2_ to anatase is influenced by the atomic layer deposition growth temperature. Specifically, at a growth temperature of 100 °C (high concentration of TDMAT precursor traces), this transition occurs at a postdeposition annealing (PDA) temperature of 375 °C.^20^ Conversely, when am.-TiO_2_ is grown at 200 °C (low concentration of TDMAT precursor traces), a gradual crystallization toward rutile is observed at 300 °C.^21^

For the ALD TiO_2_ samples grown at 200 °C, the rutile-to-anatase phase ratio was determined by differentiating the rutile (∼609 cm^–1^) and anatase (∼639 cm^–1^) Raman signal peak ratio.^22^Figure 3b interestingly reveals that the rutile-to-anatase ratio increases logarithmically with the heating rate. The rutile-to-anatase ratio reached its highest value of 90% for the 200 °C grown RTA sample. These results indicate that the phase composition can be controlled by the heating ramp rate, even when the target temperature and time are fixed.

The high degree of oxide defects within the TDMAT-free 200 °C grown TiO_2_ facilitates direct amorphous to rutile crystal nucleation.^18^ Because of oxygen vacancies, diffusion of oxygen to the material must precede the crystallization. Therefore, we propose oxygen diffusion kinetics to explain the difference in the rutile-to-anatase ratio. For fast heating rates, oxygen diffusion through the material is fast, forming oxygen-rich regions favoring the formation of the most stable rutile phase. On the contrary, for slow heating rates, a less stable anatase phase can form under oxygen-deficient regions.

Next, we investigated the photocatalytic performance of SiO_2_–TiO_2_ core–shell particles grown at different temperatures (100 and 200 °C) and subsequently annealed at 500 °C, with varying heat ramping rates. Our study was focused on evaluating their potential to enhance the hydrogen production reaction from an aqueous solution containing methanol as a sacrificial agent. Methanol acts as an electron donor, capturing photogenerated holes during the solar water splitting (SWS) reaction, thereby preventing the recombination of electron–hole pairs.^23,24^ Thus, more electrons are accessible for the water reduction half-reaction, enhancing the H_2_ production efficiency.

**Figure 4:**
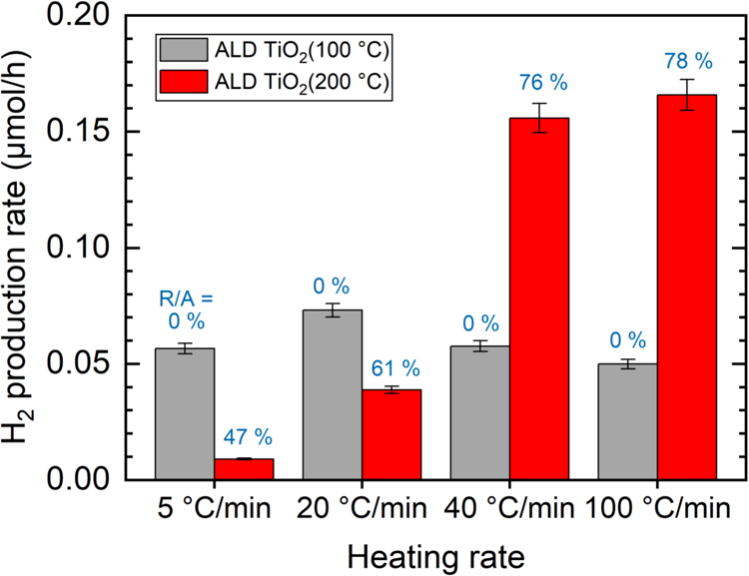
Photocatalytic hydrogen evolution test. Error bars represent the calibration error of GC.

The results presented in Figure 4 revealed that the catalyst grown at 100 °C (anatase TiO_2_) exhibited similar activity for all the heating rates. In contrast, the catalyst prepared at 200 °C (rutile-rich mixed-phase TiO_2_) displayed a strong dependence of activity on the heating rate. The activities of mixed-phase samples prepared by slow heating ramps (5 and 20 °C/min) were smaller than anatase samples, whereas the activities of mixed-phase samples prepared by fast heating ramps (40 and 100 °C/min) outperformed other samples by a clear margin. In conclusion, the best photocatalytic activity is obtained for mixed-phase TiO_2_ with >70% rutile-to-anatase ratios.

Mixed-phase TiO_2_ is often prepared from anatase-rich TiO_2_ nanoparticles, such as P25, by increasing the calcination temperature (e.g., 500–900 °C)^25^ or the calcination duration (e.g., 4–80 h at 600 °C)^26^ in the vicinity of anatase-to-rutile phase change temperature. Changes in the calcination temperature or holding time can result in different surface areas via agglomeration or sintering of nanoparticles and, therefore, affect the apparent activity. Here, we show that starting from an amorphous TiO_2_ shell on quartz microparticles, the rutile-to-anatase ratio can be controlled, given that the ALD process is optimized by the heating rate while keeping the calcination temperature and holding time fixed.

Introduction of the anatase–rutile phase junction is beneficial to the photocatalytic activity of TiO_2_.^27^ Starting from P25 TiO_2_, Xu et al. synthesized a TiO_2_ photocatalyst with increasing rutile-to-anatase ratio by increasing the calcination temperature and found the maximum photocatalytic activity for a sample containing a 74% rutile phase.^25^ They concluded that photocatalytic activity increases with the amount of anatase–rutile phase boundaries, which is a viable explanation of our result also. We have shown that the TiO_2_ photocatalyst with an optimum rutile-to-anatase ratio can be obtained at temperatures as low as 500 °C by controlling the heating rate when TiO_2_ is fabricated by an optimized ALD process. ALD growth temperature, on the other hand, strongly affects the concentration of precursor traces that can significantly affect the anatase-to-rutile phase change temperature.

## Conclusions

In the present study, we have synthesized a particulate TiO_2_ photocatalyst by ALD using SiO_2_ particles as the support material. We investigated the effect of growth temperature on crystallization kinetics. The desorption of ALD precursor traces, pronounced for low growth temperatures, substantially increases the activation energy for TiO_2_ crystallization compared to precursor trace-free ALD TiO_2_ that can be fabricated at higher temperatures. Low-impurity ALD TiO_2_ crystallizes into mixed-phase TiO_2_, for which the phase composition was found to be sensitive to the heating ramp rate even when the heating temperature and time were fixed. The photocatalytic activity was found to be sensitive to the ALD growth temperature and heating ramp rate. The activity was observed best for mixed-phase TiO_2_ containing a high rutile-to-anatase ratio. Most importantly, these results provide insights into the role of ALD precursor traces on the crystallization mechanism and material performance in photocatalytic applications. The work also demonstrates the use of ALD, more commonly applied to fabricate thin films on planar substrates, in the fabrication of particulate photocatalysts.

## Abbreviations

TiO_2_: titanium dioxide
ALD: atomic layer deposition
am.-TiO_2_: amorphous titanium dioxide
SiO_2_: silicon dioxide
